# Production of Aluminum AA6061 Hybrid Nanocomposite from Waste Metal Using Hot Extrusion Process: Strength Performance and Prediction by RSM and Random Forest

**DOI:** 10.3390/ma14206102

**Published:** 2021-10-15

**Authors:** Muntadher Sabah Msebawi, Zulkiflle Leman, Shazarel Shamsudin, Suraya Mohd Tahir, Che Nor Aiza Jaafar, Azmah Hanim Mohamed Ariff, Nur Ismarrubie Zahari, Abdallah Abdellatif

**Affiliations:** 1 Department of Mechanical and Manufacturing Engineering, Faculty of Engineering, Universiti Putra Malaysia, Serdang 43400, Malaysia; su_mtahir@upm.edu.my (S.M.T.); cnaiza@upm.edu.my (C.N.A.J.); azmah@upm.edu.my (A.H.M.A.); rubie@upm.edu.my (N.I.Z.); 2 Advanced Engineering Materials and Composites Research Centre, Faculty of Engineering, Universiti Putra Malaysia, Serdang 43400, Malaysia; 3 Sustainable Manufacturing and Recycling Technology, Advanced Manufacturing and Materials Centre (SMART-AMMC), Universiti Tun Hussein Onn Malaysia, Batu Pahat 86400, Malaysia; shazarel@uthm.edu.my; 4 Department of Electrical Engineering, Faculty of Engineering, University of Malaya, Kuala Lumpur 50603, Malaysia; abdallahh950@hotmail.com

**Keywords:** AA6061, hot extrusion, hybrid composites, nano-CuO, nano-SiO_2_, random forest

## Abstract

To date, various studies have analysed the effects of reinforced ceramic on the properties of AA6061 recycled aluminum alloy chips, such as the tensile strength and fractography. However, a comprehensive analysis of the properties of hybrid composite with the addition of nano-silica oxide and nano-copper oxide reinforcements is still very limited. Therefore, this study aimed to optimise the factors comprising the preheating temperature (PHT), preheating time (PHti), and volume fraction (VF) of reinforcements then determine their impacts on the physical and mechanical properties of the recycled solid-state extruded composite aluminum chips. A total of 45 specimens were fabricated through the hot extrusion technique. The response surface methodology (RSM) was employed to study the optimisation at a PHT range of 450–550 °C with PHti of 1–3 h and VF of 1–3 vol% for both reinforcements (SiO_2_ and CuO). Moreover, a random forest (RF) model was developed to optimize the model based on a metaheuristic method to improve the model performance. Based on the experimental results the RF model achieve better results than response surface methodology (RSM). The functional quadratic regression is curvature and the tested variable shows stable close data of the mean 0 and α2. Based on the Pareto analysis, the PHT and VF were key variables that significantly affected the UTS, microhardness, and density of the product. The maximum properties were achieved at an optimum PHT, PHti, and VF of 541 °C, 2.25 h, 1 vol% SiO_2_ and 2.13 vol% CuO, respectively. Furthermore, the morphological results of the tensile fractured surface revealed the homogenous distribution of nano-reinforced CuO and SiO_2_ particles in the specimens’ structure.

## 1. Introduction

Composite materials have useful properties due to their constituent matrix and reinforced materials [1,2]. One of the latest generations of composites to date is the heterogeneous hybrid composite that comprises at least three distinct components or phases with various shapes and compositions.

The conventional approach for aluminum production from ores requires approximately 113 GJ per tonne of aluminum, while the secondary fabrication and production from conventional aluminum recycling methods from scrap requires around 13.6 GJ per tonne of energy [3,4]. In contrast, the production of recycled aluminum can save up to 88% of the energy used in extracting aluminum from ores [3]. However, due to the difference in properties, the recycled aluminum alloy chips are not frequently used in different applications, such as the automotive industry. The properties of recycled aluminum can be significantly improved through the utilisation of reinforced materials. The successful application of reinforcements to achieve the desired properties, including the tensile strength, with an increase in yield was recently reported [4].

Solid-state recycling is used to convert the metallic scraps into bulk material, consequently eliminating the remelting process that is commonly found in the conventional recycling approach [5,6,7]. Moreover, solid-state recycling via the hot extrusion process produces less waste and has lower environmental implications [8].

The quality and properties of hot extruded aluminum alloy chips are determined by several extrusion factors, such as process temperature, extrusion ratio, die geometry, chip morphology, and extrusion speed. These factors play a vital role in determining the final properties and microstructure of the extruded products [9]. The formation of hybrid composites with the addition of two different reinforcing ceramic particles in the aluminum matrix improved the mechanical properties [10]. The use of hybrid ceramic reinforcements was also employed to produce cheaper final products [11].

Previously, aluminum silica oxide (Al-SiO_2_) nanocomposites prepared by powder metallurgy and subsequent hot extrusion resulted in an enhanced tensile strength and compressive mechanical properties [12,13,14]. In other studies, the addition of copper oxide (CuO) reinforcement reduced the energy consumption by changing the preheating temperature to 550 °C and preheating time to 3 h for optimum mechanical properties [15,16] and reduced melting point [17]. The tribological properties are considered to be one of the major factors controlling the performance and mechanical properties of composites [18,19]. A good balance between the mechanical properties, thermal properties, and production costs are the key performance indicators for the successful development of the hybrid composites. However, incorporating CuO and silica oxide SiO_2_ nanoparticles into the aluminum matrix was difficult due to the agglomeration phenomena in the nanoparticle-reinforced metal matrix composite MMCs [20].

The mechanical properties of the composite materials could be optimised through the development of an efficient model based on machine learning. Currently, algorithms are widely used in tree-based machine learning for different applications, such as agricultural processes [21], the transportation sector [22], materials science research [23], and energy processes [24]. Tree-based algorithms are well-known and studied for the production and prediction of practical and convenient performance end results [25,26]. These algorithms are used effectively by maintaining the interactions automatically, even in the presence of various large coefficients [26]. The Random Forest (RF) model is an effective tree-based ensemble technique to carry out regression and classification studies.

This study analysed the effects of Preheating Temperature (PHT), Preheating Time (PHti), and Volume Fraction (VF) of nano-silica (nano-SiO_2_) and nano-copper oxide (nano-CuO) on the Ultimate Tensile Strength (UTS) of recycled aluminum composite. The Response Surface Methodology (RSM) technique was employed to analyse the effects of these factors. In addition, the developed Random Forest (RF) model was utilised to predict the mechanical properties of the final products. Besides evaluating the RF model using the confirmation test, the Particle Swarm Optimisation (PSO) was used to fine-tune the hyper-parameter of the RF model and improve the accuracy of the model. The RSM results were then validated and compared to those obtained from the experimental results. The morphological characterisation was carried out using the Scanning Electron Microscope (SEM) and X-ray Diffraction (XRD).

## 2. Materials and Methods

### 2.1. Fabrication of Hybrid Aluminum Nanocomposite

AA6061 aluminum alloy chips were synthesised using a block of bulk aluminum with a computer numerical control CNC (Mazak 510C) milling machine United Kingdom, at Advanced Machining Laboratory, UTHM, which produces aluminum chips from the aluminum block without affecting the properties of the recycled products [27]. The chips dimensions were obtained using the toolmaker measuring microscope, Shenzhen, China. Each particle has a measurement of 3.40–3.70 mm × 1.630 mm × 0.094 mm (length × width × thickness) with approximately 12.35 mm^2^ of surface area. The particle size of the high purity SiO_2_ and CuO reinforcement nanoparticles was 20 nm.

**Figure 1:**
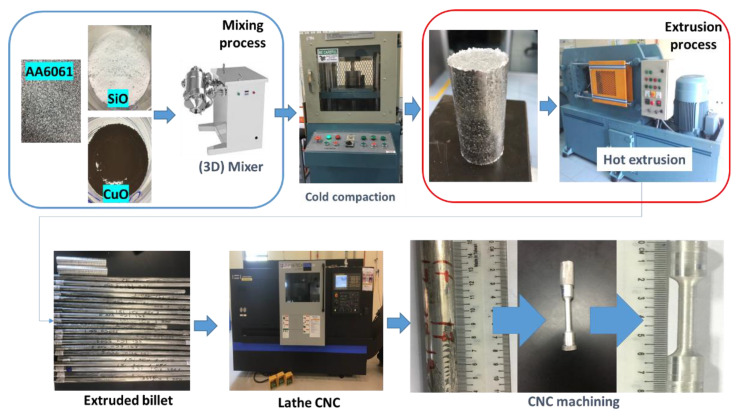
Process sequence of billet life.

The chips were first cleaned thoroughly using the ultrasonic method based on the ASTM G131-96 standards to remove any impurities and dust particles. After cleaning, the chips were mixed with nano-SiO_2_ and nano-CuO particles using the three-dimensional (3D) mixer (SYH-15). The mixture was transferred into a cylindrical shape die using the cold-pressing technique to produce a billet of approximately 90 mm × 30 mm (length × diameter, Ø). The process parameters to produce the billet are shown in Figure 1.

The billet was passed through the extrusion process with pre-designated parameters and conditions, as shown in Table 1. The parameters for PHT, PHti, and VF were varied between 450–550 °C, 1–3 h, and 1–3%, respectively. The temperature limit was set to 550 °C to avoid the formation of hot cracks at the surface of the final extruded products [28]. A ceramic heater (SOV140B), was employed to achieve the required temperature. In addition, a graphite-based lubricant was applied to the inner surface of the extrusion die and cylindrical container during the extrusion process to avoid the increase of the extrusion load due to frictional forces. The final extruded products were machined to prepare tensile test samples according to the ASTM E8/E8M-15a standard.

### 2.2. Response Surface Methodology (RSM)

The Response Surface Methodology (RSM) through the Design of Experiments (DOE) was employed to analyse the key parameters affecting the tensile strength of the extruded products. Given that RSM provides an appropriate direction required to optimise the UTS, the number of experimental runs was obtained to optimise the most suitable parameters expected to influence the response. Two replicates were included to analyse the effects of PHT, PHti, and VF of CuO and SiO_2_ on the tensile strength. Three centre points were involved in the full factorial design to examine the curvature effect. Then the RSM model (Equation (1)) suggested the adequacy and capability of the linear model in defining the relationship between the process factors over the response or otherwise.

(1) $$y=b_{0}+b_{1}X_{1}+b_{2}X_{2}+\cdots +b_{k}X_{k}$$

In the regression equation, the letter *y* is the response variable, *b*_0_ is the constant, *b*_1_, *b*_2_, …, *b_k_* are the coefficients and *X*_1_, *X*_2_, …, *X_k_* are the values of the terms. The interactions between different factors were also investigated. The factors considered and associated ranking levels during the full factorial design are presented in Table 2.

### 2.3. Random Forest Model

The Random Forest (RF) model consists of multiple decision trees (*h*) that are used and developed with the help of a random subset of different variables that categorise and replace the independent original data sets [32]. With the use of binary partitions known as splits on different variables, the DT is isolated in the predictor space. The tree “root” node constitutes and forms the comprehensive predictor space. The non-split nodes are referred to as the “terminal nodes” and form the final partition of the predictor space. According to the value of one of the predictor variables, each non-terminal node splits into two left and right descendant nodes. The data is separated into the two descendant nodes once a split had been chosen, and each of these nodes is classified in the same way as the original node. RF is an assimilated classifier, which is made up of a group of DT classifiers and is expressed as Equation (2):

R = *h* (*x*., θ_k_); k = 1,2, … (2)

where θ_k_ represents the random vector that observes identical and independent distribution and K represents the number of DT in an RF [33]. Each DT classifier defines the optimum classification result by voting in the case of a given independent variable *x*.

The RF model applied in this study consisted of 100 separate DT estimators. The Mean Square Error (*MSE*) was used in each DT to measure the quality of the split. The minimum number of samples needed to split an internal node and to be at a leaf was set to 2 and 1, respectively [34]. The maximum depth of all trees was selected to none (nodes keep expanding until the leaves contains less than min sample split). The mentioned hyperparameters are the default RF parameters used in Scikit-learn. The hyper-parameter optimisation was performed to model and reach the ultimate performance. The RF model was also implemented with the use of the Scikit-learn framework in Python.

#### 2.3.1. Particle Swarm Optimisation (PSO)

Particle Swarm Optimisation (PSO) is an important set of evolutionary algorithms commonly employed in optimisation issues and problems. PSO algorithms have been frequently used in biological population studies to analyse individual and social behaviours [35]. PSO has been used and successfully employed to enable the groups of particles, known as a swarm to traverse the search space in a semi-random manner. PSO algorithms can also determine and identify the optimum solutions by sharing information with individual particles groups. In PSO analysis, a group of *n* particles within a swarm S (Equation (3)) is used, while each particle of *S_i_* is represented by a vector [36]:

S = {S_1_, S_2_, … S*_n_*}, S is a vector represented by = {*x_i_*, *v_i_*, *p_i_*} (3)

where *x_i_* represents the current position, *v_i_* represents the current velocity, and *p_i_* shows the well-known best position within the swarm. After the position and velocity for each particle were identified, the current position and recorded position were evaluated and analysed with the performance score. In the next and upcoming iteration, the velocity *v_i_* for each particle was altered based on the previous position *p_i_* and the current global optimal position *p*, as defined in the following Equations (4) and (5):

*v_i_*^k+1^ = ω*v_i_*^k^ + c_1_r_1_(*p_i_* − *x_i_*^k^) + c_2_r_2_(*p* − *x_i_*^k^) (4)

*x_i_*^k+1^ = *x_i_*^k^ + *v_i_*^k+1^ (5)

where ω represents the inertia factor, c_1_ and c_2_ are the acceleration constants, r_1_ and r_2_ represent the random numbers in the interval of [0, 1], and k represents the iteration numbers. The above processes were used until convergence or termination constraints was achieved.

#### 2.3.2. RF Hyper-Parameter Optimisation and Evaluation

An important part of the process for the development of the RF model was measuring the model accuracy in order to analyse the performance and predictions. The Mean Squared Error (*MSE*), Mean Absolute Error (*MAE*), and correlation coefficient (*R*^2^) were primarily used during the regression analysis to determine the rate of prediction error and efficiency. The *MSE* describes the difference between both original and predicted values that were calculated and derived from the square average difference in the data set. In addition, the *MAE* provides the discrepancy and difference within the original and predicted values due to the average absolute difference in the data set. The value of *R*^2^ explains the accuracy of the matched values with the original values. The higher value of *R*^2^ from a scale between 0 and 1 indicates a good RF model. The fitting error during the prediction and training analysis in the developed model was validated and tested through *MSE*, *MAE*, and *R*^2^, which were calculated through Equations (6)–(8).

(6) $$\mathrm{MSE}=\frac{1}{N}\sum_{i=1}^{N}{\left(y_{i}-\hat{y}\right)}^{2}$$

(7) $$\mathrm{MSE}=\frac{1}{N}\sum_{i=1}^{N}|{\hat{y}}_{i}-\hat{y}|$$

(8) $$R^{2}=1-\frac{\sum_{i=1}^{N}{\left(y_{i}-{\hat{y}}_{i}\right)}^{2}}{\sum_{i=1}^{N}{\left(y_{i}-\bar{y}\right)}^{2}}$$

The performance metric, evaluation, and analysis methods were carefully configured. For various experiment through the data sets, the 10-fold cross-validation was used to evaluate the performance of the model. For each experiment, the optimum RF model architecture having the lowest *MSE* and the optimum hyper-parameter configuration would be returned. The hyper-parameter configurations are presented in Table 3.

Parameter tuning in machine learning models is very important in order to obtain desired and required results. Through PSO optimisation, the hyper-parameters were tuned in such possible ways to obtain the best results during the use of the RF model during experimentation. The values for tuned hyper-parameters in the RF model, the random state guaranteed the generation of splits that were reproducible. The random state was applied in the train test split to provide a similar set of train and test data points every time and would not assist during debugging problems and issues.

Although the generation of more trees yielded better results, certain issues and limitations were still present. The time complexity of RF improved through the selection of various large numbers of trees. An adequate estimator’s value was required for better results. The max depth of a tree is known as the largest and longest path between the leaf and the root node. The parameter that formed the DT in RF to split the minimum number of observations required for any given model is known as the min sample split. The hyper-parameter result is shown in Table 4.

## 3. Results and Discussion

### 3.1. Comparison between Single and Hybrid Nano-Reinforcement

**Figure 2:**
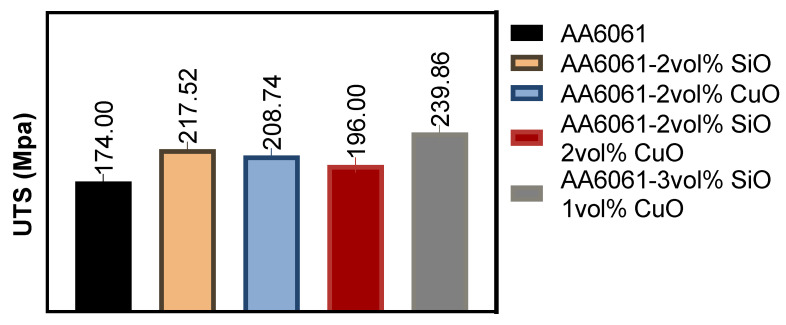
UTS of various composite samples.

According to Figure 2, the SiO_2_ reinforcement in the aluminum alloy chips improved the UTS by 22%, while the CuO reinforcement improved the UTS by 16%. However, the addition of 2 vol% SiO_2_ and 2 vol% CuO improved the UTS by 11% only. It was important to note that the UTS of a composite depends on various factors, such as reinforcement particle size, dispersion, morphology, reinforcement vol%, process parameter, and heat treatment. The RSM process was conducted using the full factorial design, which produced 35 runs followed by 10 runs. The suggested sequence was used in conducting each experiment. The maximum UTS of 239.86 MPa was obtained at PHT, PHti, and VF of 550 °C, 1 h, and 1 vol% of CuO and 3 vol% of SiO_2_, respectively. The UTS of the nanocomposite was significantly improved with the addition of reinforcements compared to pure aluminum alloy chips. As a result of the maximum PHT and the minimum VF of CuO and SiO_2_, the recycled nature of the aluminum chips and the addition of reinforcements improved the UTS of the fabricated composite from the AA6061 alloy.

**Figure 3:**
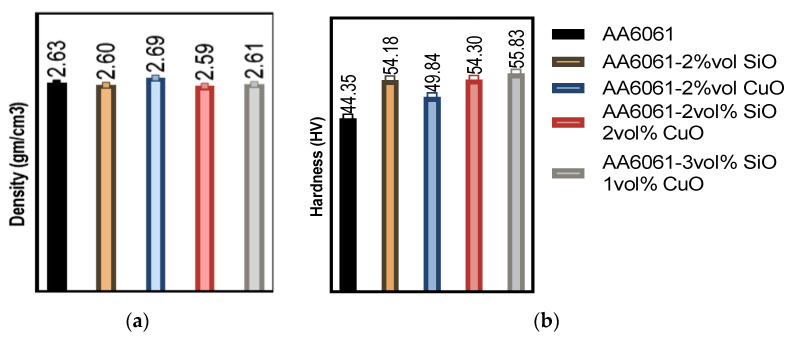
Composite samples, (**a**) density and (**b**) hardness.

The least UTS of 127.91 MPa was obtained at PHT, PHti, and VF of 450 °C, 1 h, and 1 vol% of CuO and 1 vol% of SiO_2_, respectively. This was a clear indication that lower PHT and lower VF of the reinforcements were significant towards the tensile strength of the recycled solid-state extruded AA6061 aluminum alloy chips. Although the addition of CuO was supposed to increase the density, instead, the density decreased due to the rise of SiO_2_ in the composite powder [14], as shown in Figure 3a. The reduction was compensated with the high sintering temperature since the activation energy required to drive the sintering mechanism leads to the neck growth as a result of the surface and volume diffusion.

The microhardness of the material, which refers to the hardness of the composite, increased with the temperature increase due to the reduction in the nano-reinforcement [37] and the fine grain size of the constituents. In addition, the SiO_2_ and CuO also improved the microhardness [17,38,39], as shown in Figure 3b. Pure aluminum alloy chips have a low hardness due to the non-uniform size of the chips, which was found to be the main reason behind the fluctuating values in some of the experimental results. These results indicated after produced hybrid composite, the uniform and homogenous dispersion of reinforcement particles, development of strong interfacial properties between matrix and reinforcement material, which led to the uniform distribution of loads in the composite samples to improve these mechanical properties.

### 3.2. Response Surface Methodology (RSM) for UTS

**Figure 4:**
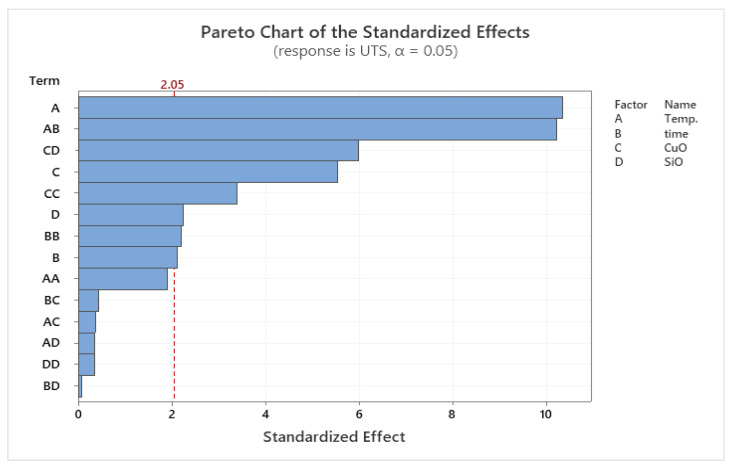
Pareto chart for UTS.

Following the interpretations of the RSM, it was obvious that the list of key parameters on the investigated factors affecting the UTS of extruded samples was PHT and VF. SEM showed the sources of variance from Table 5, displaying the *p*-value for the liner model, temperature, CuO, and SiO_2_ was significant, but the lack of fit was not significant. On the list of factors, these factors were indicated by *p* < 0.05, as shown in the Pareto chart in Figure 4. Other parameters, such as PHti (B), the combination of the PHT and PHti (AB), were insignificant. In addition, the mixture of PHT, PHti, and VF (ABC) was also insignificant towards the UTS [40].

After eliminating the non-significant terms, the final regression model to predict the ultimate tensile stress of the developed reinforced nanocomposite was expressed using Equation (9) below:

Microhardness = 797 − 4.39Temp. + 281.5Time + 105.4CuO + 17.2SiO_2_ + 0.00566Temp. × Temp. − 16.37Time × Time − 25.27CuO × CuO + 2.59SiO_2_ × SiO_2_ − 0.4277Temp. × Time + 0.0152Temp. × CuO − 0.0148Temp. × SiO_2_ + 0.92Time × CuO + 0.15Time × SiO_2_ − 12.51CuO × SiO_2_ (9)

**Figure 5:**
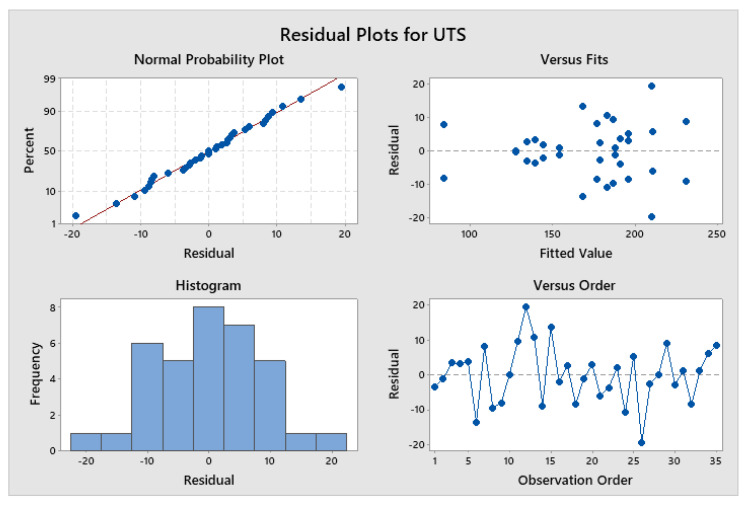
Response analysis of design of experiment for UTS.

According to the regression coefficients and analysis of variances Figure 5, the quadratic model suggested the explanation of the effects of PHT and the mixing between SiO_2_ and CuO volume fraction to analyse the UTS for the extruded composite samples. Moreover, the RSM indicated various processing parameters (PHT (A), PHT*, PHti (AB), VF (SiO_2_)*, VF (CuO) (CD), VF (CuO) (C), VF (CuO)*, VF (CuO) (CC), VF (SiO_2_), and PHti (BB)) were important. The overall quality of the model was analysed and evaluated through the results of *R*^2^, adjusted *R*^2^ (adj. *R*^2^), predicted *R*^2^ (pred. *R*^2^), and proper, precise values. The quadratic model was obtained along with mathematical modelling with a strong and accurate determination of *R*^2^ = 92.24%, which was a better regression model fit to the research studies. The adj. *R*^2^ and pred. R^2^ values were 88.23% and 80.84%, respectively. A great agreement between the adj. *R*^2^ and pred. *R*^2^ prevented the over-fitting of the mathematical model [41]. Table 6 shows the R results for UTS by RSM.

**Figure 6:**
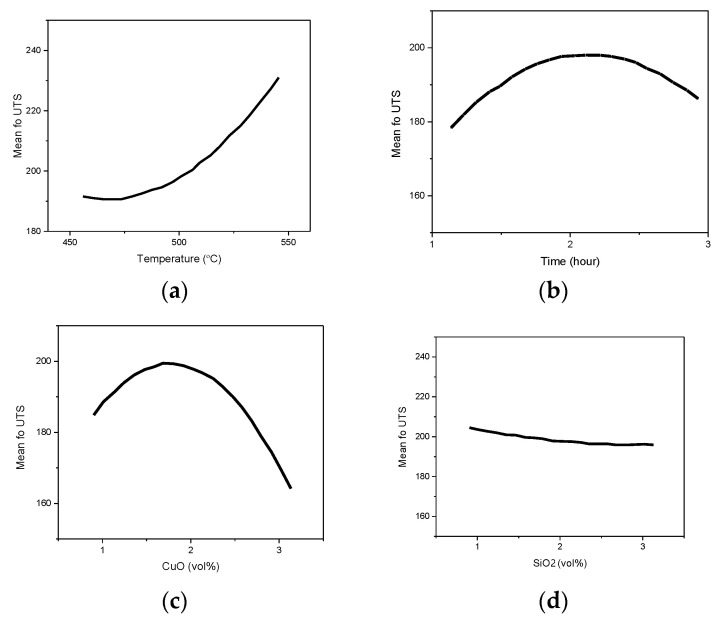
Main effects plot for UTS. (**a**) Temperature; (**b**) Time; (**c**) Copper oxide volume fraction; and (**d**) Silica oxide volume fraction.

From the microstructural point of view, a higher temperature was a requisite in obtaining a refined microstructure. The specimen extruded using the highest temperature may result in higher tensile strength. The explanation for this observed relationship was due to the fact that an increase in temperature strengthened the aluminum matrix composite AMC better by supporting the bonding of the matrix, SiO_2_, and CuO. Therefore, increasing the VF of SiO_2_ to 3 vol% and decreasing the VF of CuO to 1 vol% supported the UTS. On the other hand, the decrease of reinforcements showed that the extruded billets had the least UTS. The results presented were in agreement with the findings reported by Shazarel et al. [42]. It was obvious from the main effect plot shown in Figure 6, that the relationship between the parameters was close. All the centre points were quite close to the lines connecting the average tensile strength from low to high setting for PHT and from high to low setting for VF.

**Figure 7:**
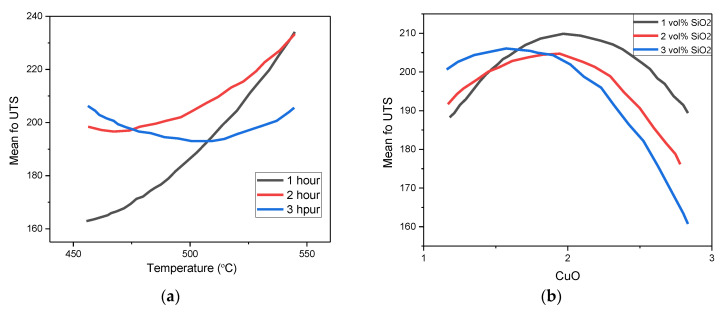
Interaction plot for UTS (**a**) Temperature vs Time; (**b**) CuO vs SiO_2_.

The plot was relevant to justify the trends observed in the experimental results and the relationship described in Figure 5 earlier. An interaction plot was developed during the analysis of the fitness of the proposed model to represent the relationship between the parameters of interest and the UTS, as shown in Figure 7.

A very similar trend was observed in the interaction plot. The model selected was sufficient in describing the effect of all the parameters on UTS. Generally, the UTS was higher when the PHT and PHti increased. On the other hand, the curvature effect was not significant, indicating that the level of interference of unwanted factors on the results reported was negligible compared to the factors captured by the presented model.

#### Optimisation of UTS

**Figure 8:**
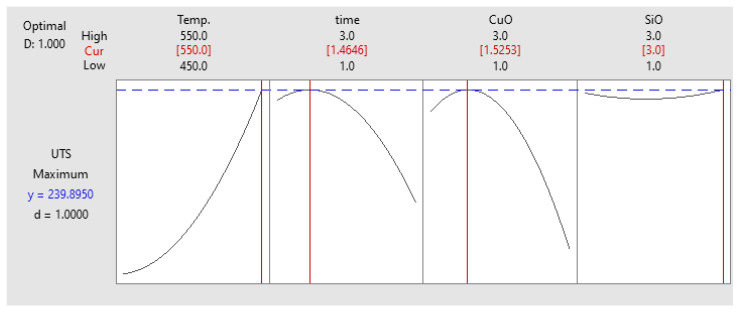
Optimisation plot for UTS.

**Figure 9:**
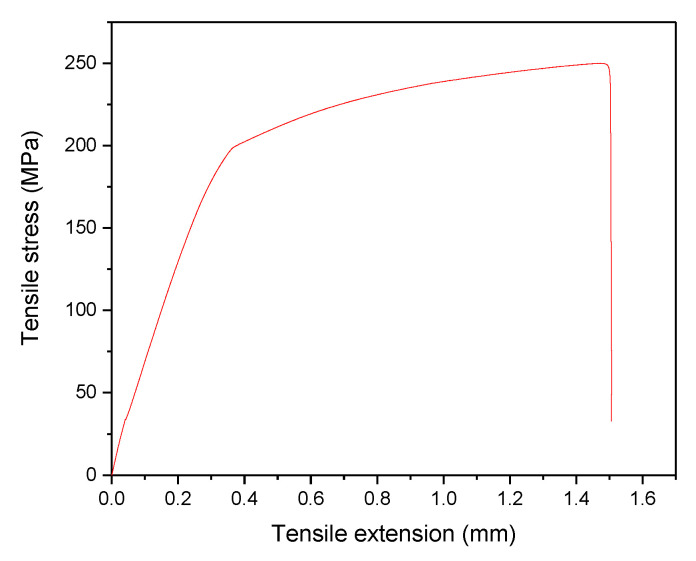
Hybrid composite optimal results.

The specific function of the response optimiser was to identify the effect of individual parameters on the UTS. The parameters from this analysis were a set of specific values in terms of all the parameters that were useful to obtain the optimum UTS. The optimum value for UTS from the response optimiser approach is presented in Figure 8. The resultant composite specimen, according to the optimised PHT, PHti, and VF of 550 °C, 1.46 h, and 1.52 vol% CuO and 3 vol% SiO_2_, respectively, was used to validate the developed quadratic regression model, as shown in Figure 9. The value of the experimental UTS for the specimen was 245.51 Mpa.

**Figure 10:**
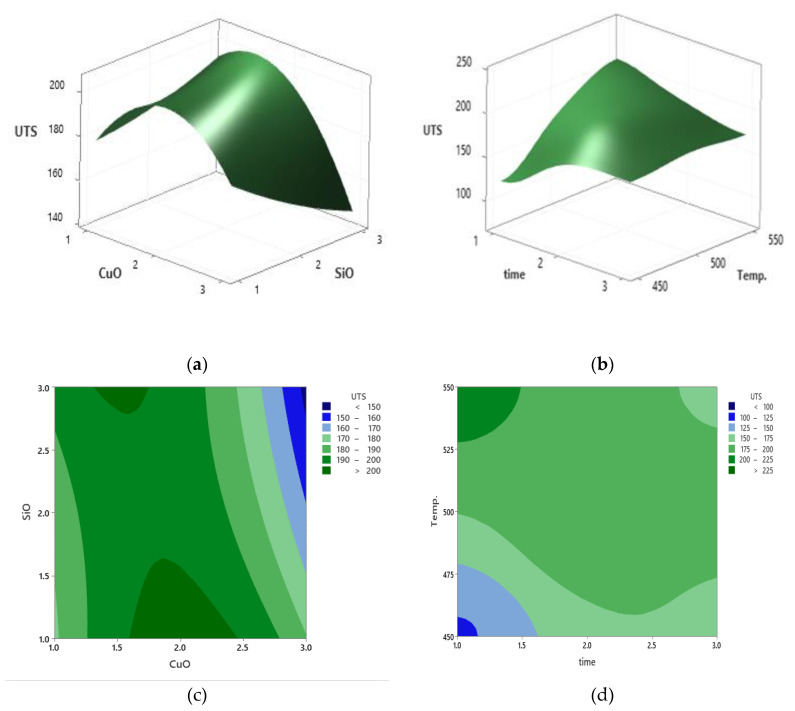
(**a**) Response surface plots of UTS vs. SiO_2_ and CuO. (**b**) Response surface plots of UTS vs. temp, and time. (**c**) Contour plot of UTS vs. SiO_2_ and CuO. (**d**) Contour plot of UTS vs. temp, and time.

The established relationship and the effect of the reinforcement in addition to the VF of CuO increased the UTS, which then decreased after the addition of 1.58 vol% SiO_2_. Figure 10a shows the response surfaces plot, which was further used to visualise the significance of these parameters, while Figure 10b shows the relationship and effect of the PHT and increase in PHti during the analysis of UTS. Based on the results, the UTS improved significantly due to the increase in PHT and PHti. Furthermore, Figure 10c shows the contour plot of UTS with the interaction between the CuO and SiO_2_. According to the contour plot, it can be concluded that the UTS of the composite increased after the addition of 2.75 vol% SiO_2_ and 2.25 vol% CuO. Figure 10d shows the contour plot of UTS that describes the interaction between the PHT and VF of the aluminum. It was clear that the increment of PHT increased the UTS, while the optimum UTS was obtained with 1.58 vol% CuO and 3 vol% SiO_2_. A comparable relationship was reported in the study conducted by Madeva et al. [43].

### 3.3. RSM for Microhardness

**Figure 11:**
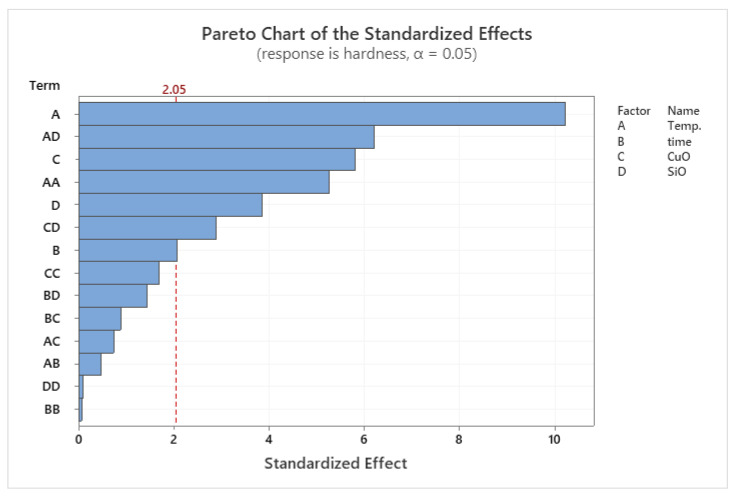
Pareto chart of the standardized effects for microhardness.

The DOE results of the microhardness are presented the model analysis proposed the determination of the levels of related experimental variables are shown in Table 7. Since the calculated *p*-value of the developed model was less than the standard (*p* < 0.05), the model was statistically significant, as described in previous literature [44,45]. The effect of PHT on the microhardness is shown in the Pareto chart in Figure 11. The model showed significant results according to the RSM of microhardness results versus PHT, PHti, and VF.

For microhardness, the values of *R*^2^, adj. *R*^2^, and pred. *R*^2^ are shown in Table 8. The *R*^2^ value of 0.9054 was favourable for this analysis and provided an excellent explanation of the relationship between the independent variables and the response. The pred. *R*^2^ of 0.8565 was within a reasonable agreement with the adj. *R*^2^ of 0.7679 [41].

The factors affecting the microhardness of composite samples were evaluated from the factorial model. The PHT was observed to have direct effects on the hardness of composite samples. The lower values of microhardness were observed at low PHT and low VF. In comparison, the high PHT and VF of SiO_2_ and CuO led to the strengthening of the composite, especially SiO_2_ that had a great effect on the microhardness. A low VF resulted in a low microhardness [46,47]. Figure 11 shows the Pareto chart of the standardised effects. The microhardness was affected mainly by PHT and the VF, while PHti only showed minor effects.

**Figure 12:**
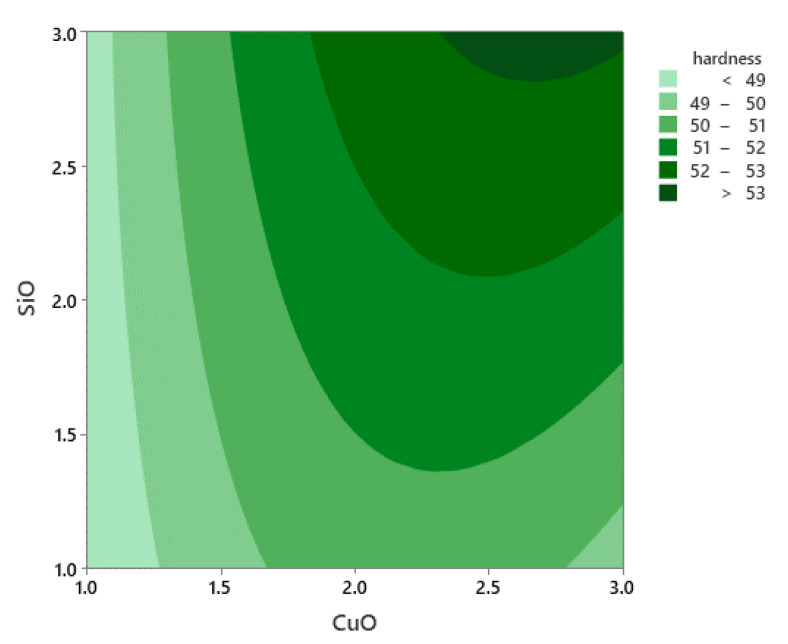
Contour plot for microhardness.

Furthermore, the contour plot for the microhardness shown in Figure 12 indicates that any difference in VF of SiO_2_ and CuO produced the highest microhardness when the temperature increased to the peak value of 521 °C. After eliminating the non-significant terms, the final regression model to predict the microhardness of the developed reinforced composite was expressed using Equation (10) below:

Microhardness = −457.5 + 2.018PHT − 2.27PHti + 8.01CuO − 17.77SiO_2_ − 0.002029PHT × PHT + 0.062PHti × PHti − 1.633CuO × CuO − 0.093SiO_2_ × SiO_2_ + 0.00260PHT × PHti − 0.00399PHT × CuO + 0.03361PHT × SiO_2_ + 0.239PHti × CuO + 0.392PHti × SiO_2_ + 0.783CuO × SiO_2_ (10)

The equation indicates that the PHT has a significant effect on the microhardness.

#### Optimisation of Microhardness

**Figure 13:**
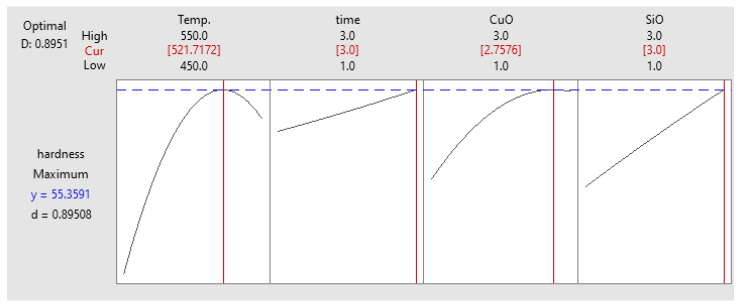
Optimisation plot for microhardness.

The optimised result was relatively close to the previous optimisation results. The maximum microhardness obtained at 58.69 HV coincided with the PHT, PHti, and VF of 521 °C, 3 h, and 2.75 vol% CuO, respectively, as shown in Figure 13. The optimised solution was consistent with the experimental results of 61.32 HV. These experimental results supported the discussion earlier related to the influence of process parameters on the response [48].

### 3.4. RSM for Density

**Figure 14:**
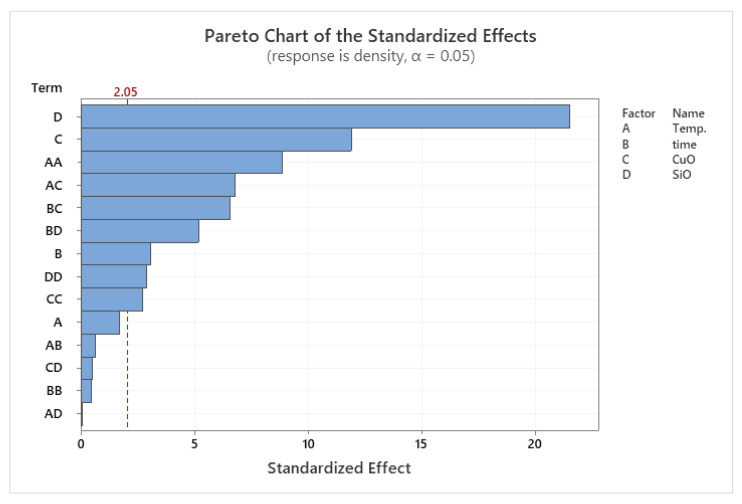
Pareto chart of the standardized effects for density.

The DOE results for the density are shown in Table 9, while the effect of the reinforcement on the density is shown in the Pareto chart in Figure 14. The model was in accordance with the RSM response results versus PHT, PHti, and VF. Since the calculated *p*-value of the developed model was less than the standard (*p* < 0.05), the model was statistically according to the desired model [44,45,49]. The model was statistically correct.

The value of *R*^2^, adj. *R*^2^, and pred. *R*^2^ for density are shown in Table 10. The *R*^2^ value of 0.9759 was favourable, which was closer to 1, and provided an excellent explanation of the relationship between the independent variables and the response. The pred. *R*^2^ of 0.9634 was within a reasonable agreement with the adj. *R*^2^ of 0.9458.

The factors affecting the density of composite samples were evaluated from the RSM model. The PHT was observed to have little to no effect on the microhardness of the hybrid nanocomposite material. The lower density was observed at very low PHT and VF. In contrast, the high PHT and VF of SiO_2_ and CuO led to the strengthening of the composite, especially SiO_2_ that had a great effect on the density. A low VF and PHT resulted in low density [46,47].

**Figure 15:**
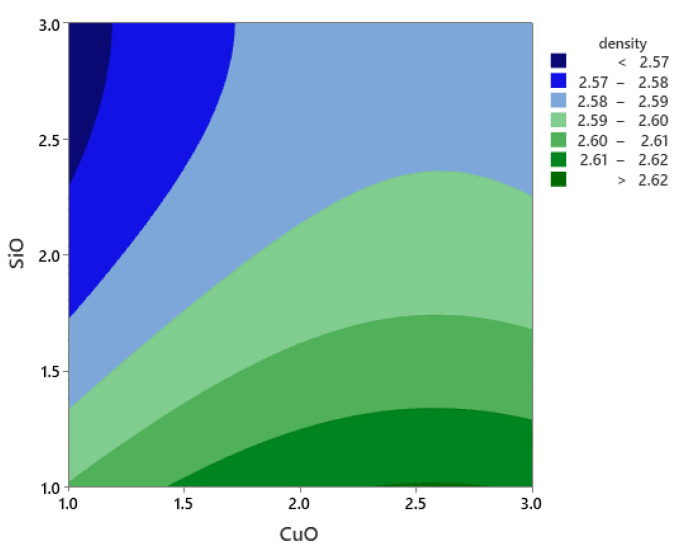
Contour plot for density.

The density was affected mainly by the VF of SiO_2_ and CuO. As shown in Figure 15, the contour plot for density indicates that the increase in VF of SiO and CuO increased the density at PHT and PHti of 500 °C and 2 h, respectively.

After eliminating the non-significant terms, the final regression model to predict the density of the developed reinforced nanocomposite was expressed using Equation (11) below:

Density = −5.076 − 0.01024PHT + 0.0112PHti + 0.1083CuO − 0.0445SiO_2_ + 0.000010PHT × PHT + 0.00137PHti × PHti − 0.00808CuO × CuO + 0.00857SiO_2_ × SiO_2_ + 0.000011PHT × PHti − 0.000113PHT × CuO + 0.000001PHT × SiO_2_ − 0.005456PHti × CuO − 0.004300PHti × SiO_2_ + 0.000425CuO × SiO_2_ (11)

The equation indicates that the PHT had a significant effect on density.

#### Optimisation of Density

**Figure 16:**
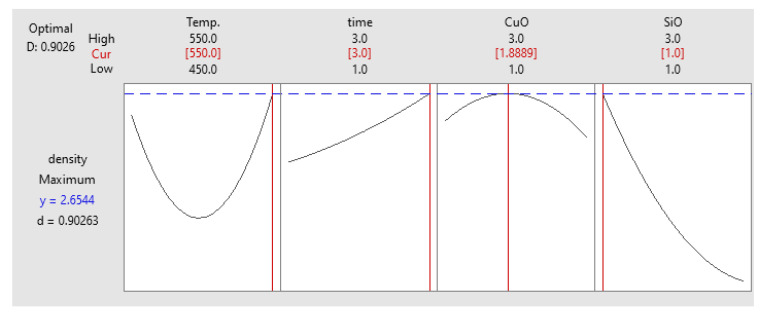
Optimisation plot for density.

Based on the full factorial optimisation, the optimised results were in accordance with previous optimisation results. The maximum microhardness obtained coincided with PHT, PHti, and VF of 265 °C, 3 h, 1 vol% SiO_2_ and 1.88 vol% CuO, respectively. As shown in Figure 16, the microhardness was 58.693 HV, which was consistent with the experimental results of 61.3266 HV. The experimental results supported the early discussion related to the influence of process parameters on the response [50,51].

### 3.5. Multi-Objective Optimisation

**Figure 17:**
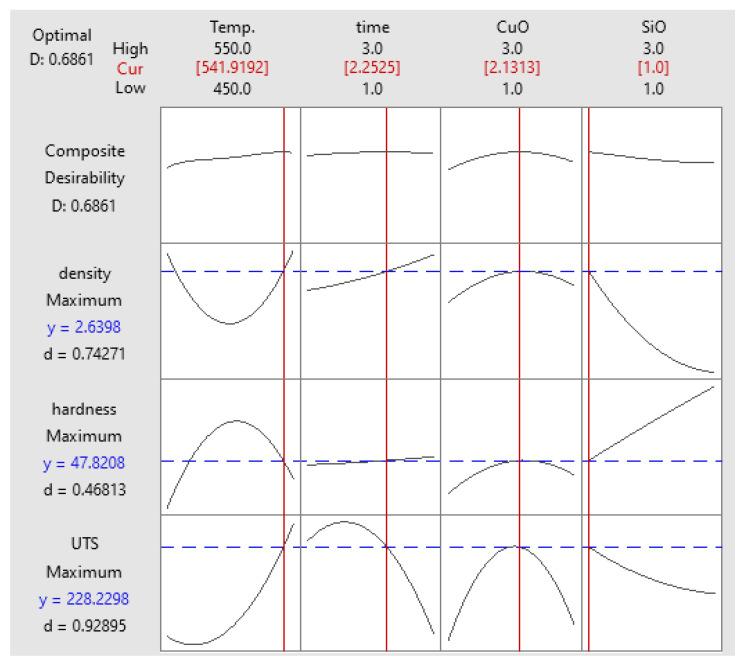
UTS, microhardness and density.

The response service methodology (RSM) was employed to optimise the parameters and obtain the maximum value of UTS, microhardness, and density. The optimised results were in accordance with the results of the previous optimisation. The maximum response obtained coincided with PHT, PHti, and VF of 541 °C, 2.25 h, and 1 vol% SiO_2_ and 2.13 vol% CuO, respectively. As shown in Figure 17, the UTS, microhardness, and density were 228.82 MPa, 47.82 HV, and 2.63 g/cm^3^, respectively [52].

### 3.6. Confirmation Test

Three confirmation tests (CT) were performed to validate the empirical results. Based on the factorial analysis, the optimum parameters for PHT, PHti, and VF. Three composite specimens were produced based on these optimum parameters to validate the developed quadratic regression RF model, in addition to the RSM model which was developed and expressed in Equations (9)–(11) respectively. Based on the confirmation test presented on Table 11, then comparison the experimental values with the predicted values from the two developed models RF and RSM of the three specimens are given in Table 12 and Table 13. The calculated errors were negligible in comparison with the obtained UTS, microhardness, and density. The calculated errors between the experimental and the optimum results were in the range of ±3%. The results successfully confirmed the reproducibility of the experimental data. The RF model outperforms the RSM model as shown in Table 13, where the RF model provides a flexible way to predict the response surface function. Moreover, RF constructs a sequence of DT, with each previous DT aiming to remove the errors of the current sequential DT model, and produces the final prediction using a weighted total of the predictions provided by the sequentially constructed DT. Also, RF provides the cross-validation technique which helps to avoid overfitting.

### 3.7. Overall RF Results for Validation and Prediction

The microhardness, UTS, and density prediction analysis and framework were modelled with higher accuracy and precision using the RF technique to avoid complex experiments and mathematical calculations. A very comprehensive and detailed comparative analysis was carried out between the model and the experiment to verify the performance. The training performance of the models was dependent on the data set under the supervision of the learning algorithms. According to the No Free Lunch (NFL) theorem, the performance and working of the RF model will not always be highly accurate and precise for each data set [53]. The four parameters comprising PHT, PHti, VF of CuO, and VF of SiO_2_ were used as the input parameters, while microhardness, UTS, and density were the output parameters to evaluate the training performance of the RF model.

Before dealing with the data, the data were normalised to fit a range of [0, 1]. Data normalising boosts the evaluation and prediction while in use. The microhardness, UTS, and density of the data samples were used in the training data, and k-fold cross-validation was set to 10. The confirmation tests were used as the test data for the verification of the accuracy of the RF model. For this model, a complete data set of 48 data samples were used. A total of 45 samples were obtained from the full factorial, while the output (microhardness, UTS, and density) was obtained from the experimental results. In addition, 45 data samples were chosen and selected at random for the training and samples validation, while the remaining 3 data samples were employed as the testing samples for output prediction assessment and the confirmation test to validate the results.

Low *MSE* and *MAE* values demonstrated better results in the RF model, as shown in Table 14. Overall, the data set, the confirmation test errors, and *MSE* and *MAE* values of the RF model were low, indicating that the model performed well in predicting the microhardness, UTS, and density. The highest *R*^2^ value for the RF model was 0.9647. Moreover, the *MSE* and *MAE* values of the RF model were lower, indicating the highly accurate results with no significant over-estimation or under-estimation of the targeted values. This study concluded the significance and efficiency of the RF model in predicting the microhardness, UTS, and density of the nano-reinforced AA6061 aluminum chips based on the *MAE* and *MSE* values with low error rate between the experimental and predicted results.

### 3.8. Fractographic Analysis of Tensile Sample

**Figure 18:**
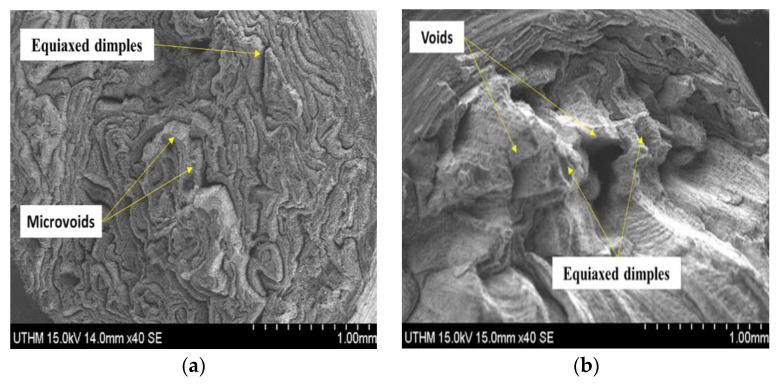

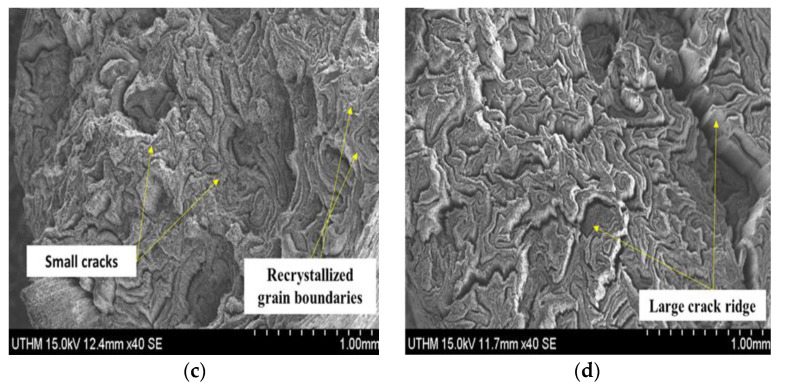
SEM micrographs of fracture surface of the tensile sample (**a**) 2 vol% CuO, 550 °C and 3 h. (**b**) 2 vol% SiO_2_, 550 °C and 3 h. (**c**) 1 Vol% SiO_2_, 1 vol% CuO, 550 °C and 3 h. (**d**) 1 vol% SiO_2_, 1 vol% CuO, 450 °C and 1 h.

The Scanning Electron Microscope (SEM) was used in analysing the morphology of fractured surface for tensile profiles of hot extruded composite samples. It was noticed that the fractured surface among tensile samples differed in contours. As shown in Figure 18a, samples extruded at PHT, PHti, and VF of 550 °C, 3 h, and 2 vol% CuO, respectively, exhibited the lowest UTS of 208 MPa, which had equiaxed dimples with microvoids causing poor material consolidation [17]. The fractographic analysis revealed improper metallurgical interface bonding at the time of extrusion. The uniformly void and equiaxed dimples cracks could easily be observed with added SiO_2_ to the alloy in PHT to 550 °C, 3 h and SiO_2_ 2 vol% with few effects on UTS of 217 MPa as shown in Figure 18b the recycled aluminum samples could have mixed through various fracture mechanisms that formed the microvoids and cleavage planes, demonstrating the effect of reinforced VF on the fractured surface.

The fractographic analysis also revealed that the weakest point served as the crack initiation site for the failure. This was an indication of the sudden change in the force equilibrium in the samples, as seen in Figure 18c. The failure was also due to the improper bonding in the extruded aluminum chips, which directly influenced the mechanical strength (127 MPa) of the extruded aluminum samples at PHT, PHti, and VF of 450 °C, 1 h, 1 vol% CuO and 1 vol% SiO_2_.

The effect of reduction in preheating temperatures from 550 °C to 450 °C was clear effected on the extruded sample microvoids and other defects and impurities served as the stress concentration sites and were responsible for the decrement in strength and other mechanical properties. The fracture surface exhibited ridges and large crack ridge instead of equiaxed dimples, as seen in Figure 18d. Such sample showed rough and large cracks, while the fracture occurred near the grip area of the sample due to the brittle failure with little plastic deformation before the fracture.

**Figure 19:**
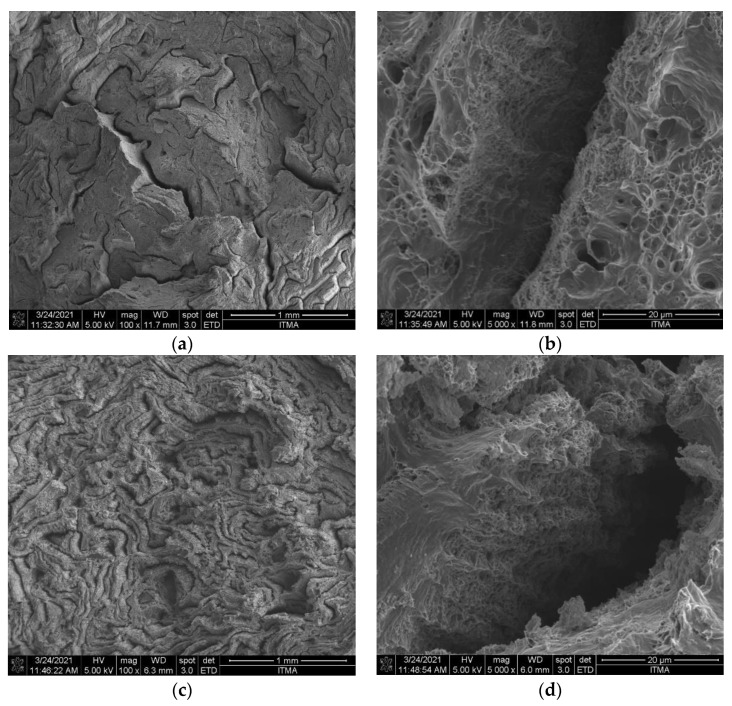
FESEM micrographs of fracture surface (**a**) AA6061 chips temp. 550 °C, time 3 h 100×, (**b**) AA6061 chips temp. 550 °C, time 3 h 5000×, (**c**) temp. 541 °C, time 2.25 h, CuO 2.13 vol%, SiO_2_ 1 vol% 100×, (**d**) temp. 541 °C, time 2.25 h, CuO 2.13 vol%, SiO_2_ 1 vol% 5000×.

The Field Emission Scanning Electron Microscope (FESEM) was used in analysing the morphology of fractured surface for AA6061 chips extruded sample at PHT, PHti, and VF of 450 °C and 3 h the presence of cleavage fractures, microvoids, and unequal dimples could easily be seen in some regions, as shown in Figure 19a,b.

In addition, the uniformly distributed dimples and small cracks could easily be observed with the increase in PHT to 541 °C and VF of CuO 2.13 vol%, SiO_2_ 1 vol%, as shown in Figure 19c,d. The non-uniform distribution of reinforcement particles led to the stress concentration site, which caused the crack initiation and subsequent failure. The high PHT ensures the uniform distribution of these reinforcement particles. The high PHT also improved the interfacial properties for a better and homogenous stress distribution. When the reinforced particles were well distributed in the aluminum matrix, the interfacial properties were significantly improved, where these improvements enhanced the mechanical properties, such as UTS and compressive strength, due to the proper distribution of stress in the composite samples. At optimal parameters, the sample exhibited the highest UTS of 239 Mpa. The positive effect of high PHT was proven in the fracture analysis. The presence of microvoids and fine dimples indicated the ductile nature of the fracture. Furthermore, clear shear and tears were observable on the fractured surface, while cleavage planes decreased significantly. The presence of a large number of small and fine dimples on the fractured surface was evidence of the influence of high PHT and low VF of reinforcements. These microvoids were formed due to the elongation of voids that filled up the available spaces to form dimples, as shown in the fractured surface. The fractography analysis further revealed that the maximum PHT and minimum VF resulted in adequate consolidation and high strength recovery of the composite material [18]. The fracture surface analysis of the optimal sample exhibited conical equiaxed dimples and the fine topography of the fracture surface in the absence of microcracks. The failure was observed along the middle part of the sample due to the ductile failure mode, which showed plastic deformation just before the final fracture.

### 3.9. X-ray Diffraction (XRD) Analysis

**Figure 20:**
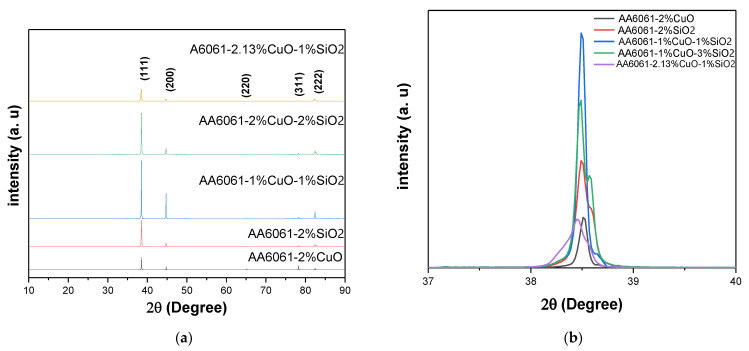
(**a**) XRD intensity pattern; and (**b**) XRD diffraction for peak (1,1,1).

Figure 20a, shown the XRD of the angle measured at 2θ in the 10–90 range. The XRD patterns of the aluminum matrix with different reinforcement concentrations. The figure shown that the laser peened sample exhibited peak broadening and a considerable shift in XRD peaks to larger Bragg’s angle that indicated the presence of microstrain and decrement of the lattice constant, which was the (hkl) spacing after the laser peening. Apart from that, the (222) and (200) planes were much less pronounced in the base material, whereas the laser peened surface showed more intense and higher diffractions from those planes [54].

The crystallinity analysis was conducted on the three most important samples utilising their highest, optimum, and lowest parameters, as presented in Figure 20b, and Table 15.

The crystalline structure was identified by matching the data and peaks with the standard JCPDS data. Based on the results, the extruded sample using the optimum parameters (541 °C, 2.25 h, 2.13 vol% CuO and 1 vol% SiO_2_, respectively) recorded the lowest intensity of almost 37,892 a.u. compared to other samples. In contrast, for the extruded sample using the optimum parameters (450 °C, 1 h, 1 vol% CuO and 3 vol% SiO_2_, respectively) and (550 °C, 3 h, 1 vol% CuO and 1 vol% SiO_2_, respectively), the intensity at the plane (111) was 130,112 and 182,419 a.u., respectively.

## 4. Conclusions

A thorough investigation on the effects of PHT, PHti, and VF of CuO and SiO_2_ on UTS, microhardness, and density of the extruded nano-reinforced aluminum composite was reported in this study. The recycled hybrid aluminum alloy chips reinforced with nano-CuO and nano-SiO_2_ were fabricated using the direct recycling method. Based on the RSM results, the PHT, PHti, and VF of hybrid reinforcements demonstrated significant influence to obtain an optimum UTS of the nano-reinforced aluminum composite. In addition, the high PHT and high VF of CuO and SiO_2_ improved and strengthen the bonding of aluminum chips through the inter-particle diffusion transport of matter and produced the highest microhardness. Furthermore, the density was affected by the hybrid reinforcements and preheating temperate with less effect for preheating time. Using the CuO as the reinforcement particle, the overall energy reduced according to the change in PHT and PHti of 550 °C and 3 h, respectively, to PHT and PHti of 541 °C and 2.25 h, respectively, to achieve the optimal properties. Based on the fractured surface analysis, the hot extrusion parameters affected the tensile strength of the specimen, while the fine topography and dimples were apparent under SEM and FESEM imaging with the increase in PHT and VF of CuO and decrease in VF of SiO_2_, based on the experimental results the RF model achieve better results than response surface methodology (RSM) and more accurate Such models could assist in predicting the outcome of the processing materials, which will be helpful to minimise and limit the number of experiments, therefore, minimising the time and energy consumption due to the high prediction rate. Moreover, this study demonstrated that the use of machine learning was significant to obtain high efficiency in research studies and manufacturing processes.

## Figures and Tables

**Table 1:** Factors used in conducting the hot extrusion.

Parameter	Value/Type
Shape of the Die	Round
Ratio used in extrusion, R	5.4
Diameter of the billet, Ø (mm)	30
Speed during extrusion, s (mm/s)	1
Container temp., (°C)	300
Die temp., (°C)	300

**Table 2:** Factors considered in the design of experiment and associated levels.

Symbol	Process Parameter	Levels			References
		Low (−1)	Center (0)	High (+1)	
A	Preheating temperature (PHT) (°C)	450	500	550	[29,30]
B	Preheating time (PHti) (hour)	1	2	3	[29]
C	Volume fraction CuO (%vol)	1	2	3	[19,31]
D	Volume fraction SiO_2_ (%vol)	1	2	3	[14]

**Table 3:** Hyperparameter configuration.

Model	Selected Hyperparameter	Type	Search Space
RF	n estimators	Discrete	[5, 50]
	Max depth	Discrete	[5, 50]
	Min samples split	Discrete	[2, 7]
	Min samples leaf	Discrete	[1, 7]
	Max features	Discrete	[1, 4]

**Table 4:** Data of tensile strength results.

Model	N Estimators	Max Depth	Min Sample Split	Min Sample Leaf	Max Feature
Random Forest	35	27	2	1	4

**Table 5:** The Analysis of Variance of UTS by RSM.

Source	DF	Adj SS	Adj MS	F-Value	*p*-Value	Effect
Model	15	48,434.2	3228.9	23.00	0.000	Significant
Blocks	1	115.5	115.5	0.82	0.372	
Linear	4	20,656.5	5164.1	36.78	0.000	
Temp.	1	15,017.1	15,017.1	106.95	0.000	Significant
Time	1	621.5	621.5	4.43	0.044	Significant
CuO	1	4311.5	4311.5	30.71	0.000	Significant
SiO_2_	1	706.4	706.4	5.03	0.033	Significant
Square	4	3680.1	920.0	6.55	0.001	Significant
Temp. × Temp.	1	504.9	504.9	3.60	0.068	
Time × Time	1	675.3	675.3	4.81	0.036	
CuO × CuO	1	1608.9	1608.9	11.46	0.002	
SiO_2_ × SiO_2_	1	16.9	16.9	0.12	0.731	
Two-Way Interaction	6	19,711.1	3285.2	23.40	0.000	Significant
Temp. × Time	1	14,635.9	14,635.9	104.24	0.000	
Temp. × CuO	1	18.4	18.4	0.13	0.720	
Temp. × SiO_2_	1	17.6	17.6	0.13	0.726	
Time × CuO	1	27.1	27.1	0.19	0.664	
Time × SiO_2_	1	0.7	0.7	0.00	0.945	
CuO × SiO_2_	1	5011.5	5011.5	35.69	0.000	
Error	29	4071.9	140.4			
Lack-of-Fit	10	1808.9	180.9	1.52	0.208	Not significant
Pure Error	19	2263.0	119.1			
Total	44	52,506.1				

**Table 6:** *R* value for UTS by RSM.

*R*-Sq	*R*-Sq (Adj)	*R*-Sq (Pred)
92.24%	88.23%	80.84%

**Table 7:** The Microhardness Analysis of Variance by RSM.

Source	DF	Adj SS	Adj MS	F-Value	*p*-Value	Effect
Model	15	651.886	43.459	18.51	0.000	Significant
Blocks	1	85.364	85.364	36.35	0.000	
Linear	4	369.472	92.368	39.33	0.000	
Temp.	1	245.486	245.486	104.54	0.000	Significant
Time	1	9.986	9.986	4.25	0.048	Significant
CuO	1	79.224	79.224	33.74	0.000	Significant
SiO_2_	1	34.775	34.775	14.81	0.001	Significant
Square	4	163.133	40.783	17.37	0.000	Significant
Temp. × Temp.	1	64.799	64.799	27.59	0.000	
Time × Time	1	0.010	0.010	0.00	0.949	
CuO × CuO	1	6.721	6.721	2.86	0.101	
SiO_2_ × SiO_2_	1	0.022	0.022	0.01	0.924	
2-Way Interaction	6	118.504	19.751	8.41	0.000	Significant
Temp. × Time	1	0.539	0.539	0.23	0.636	
Temp. × CuO	1	1.277	1.277	0.54	0.467	
Temp. × SiO_2_	1	90.346	90.346	38.47	0.000	
Time × CuO	1	1.828	1.828	0.78	0.385	
Time × SiO_2_	1	4.913	4.913	2.09	0.159	
CuO × SiO_2_	1	19.601	19.601	8.35	0.007	
Error	29	68.100	2.348			
Lack-of-Fit	10	35.669	3.567	2.09	0.080	Not significant
Pure Error	19	32.431	1.707			
Total	44	719.986				

**Table 8:** *R* value for microhardness by RSM.

*R*-Sq	*R*-Sq (Adj)	*R*-Sq (Pred)
90.54%	85.65%	76.79%

**Table 9:** The Density Analysis of Variance by RSM.

Source	DF	Adj SS	Adj MS	F-Value	*p*-Value	Effect
Model	15	0.025872	0.001725	78.13	0.000	Significant
Blocks	1	0.000218	0.000218	9.89	0.004	
Linear	4	0.013605	0.003401	154.07	0.000	
Temp.	1	0.000064	0.000064	2.89	0.100	Not significant
Time	1	0.000207	0.000207	9.36	0.005	Significant
CuO	1	0.003124	0.003124	141.51	0.000	Significant
SiO_2_	1	0.010210	0.010210	462.52	0.000	Significant
Square	4	0.003494	0.000873	39.57	0.000	Significant
Temp. × Temp.	1	0.001726	0.001726	78.17	0.000	
Time × Time	1	0.000005	0.000005	0.22	0.646	
CuO × CuO	1	0.000164	0.000164	7.44	0.011	
SiO_2_ × SiO_2_	1	0.000185	0.000185	8.39	0.007	
2-Way Interaction	6	0.002574	0.000429	19.43	0.000	Significant
Temp. × Time	1	0.000009	0.000009	0.41	0.527	
Temp. × CuO	1	0.001015	0.001015	45.97	0.000	
Temp. × SiO_2_	1	0.000000	0.000000	0.01	0.941	
Time × CuO	1	0.000953	0.000953	43.15	0.000	
Time × SiO_2_	1	0.000592	0.000592	26.80	0.000	
CuO × SiO_2_	1	0.000006	0.000006	0.26	0.613	
Error	29	0.000640	0.000022			
Lack-of-Fit	10	0.000251	0.000025	1.23	0.334	Not significant
Pure Error	19	0.000389	0.000020			
Total	44	0.026512				

**Table 10:** *R* value for density by RSM.

*R*-Sq	*R*-Sq (Adj)	*R*-Sq (Pred)
97.59%	96.34%	94.58%

**Table 11:** The confirmation tests details.

CT	Temp.	Time	CuO	SiO_2_
1	550	1.46	1.52	3
2	541	2.25	2.13	1
3	541	2.25	2.13	0.5

**Table 12:** The results obtained from the random forest and RSM tested on the confirmation test.

CT	Experimental			Predicted (RF)			Predicted (RSM)		
	UTS	Hardness	Density	UTS	Hardness	Density	UTS	Hardness	Density
1	245.51	55	2.58	243.865	54.637	2.579	238.004	49.727	2.417
2	232.66	47	2.63	226.47	46.87	2.61	225.704	47.829	2.29
3	210.04	45	2.6	212.78	45.4	2.62	232.328	46.417	2.288

**Table 13:** Random Forest and RSM Prediction Error.

CT	RF Prediction Error			RSM Prediction Error		
	UTS	Hardness	Density	UTS	Hardness	Density
CT 1	0.67%	0.66%	0.03%	3.05%	9.58%	6.31%
CT 2	2.73%	0.27%	0.76%	2.98%	1.76%	14.8%
CT 3	1.28%	0.88%	0.76%	10.61%	3.14%	12%

**Table 14:** RF performance metrics.

Model	MSE	MAE	*R*-Squared (*R*^2^)
RF	0.03717	0.13171	0.9628

**Table 15:** Crystallite size of samples at peak (1,1,1).

Specimen	2 Theta	Intensity	Crystallite (Å)
AA6061-2%CuO	38.51297	31342	91.23
AA6061-2%SiO_2_	38.51251	83249	48.72
AA6061-1%CuO-1%SiO_2_	38.49631	182419	96.77
A6061-1%CuO-3%SiO_2_	38.50309	130112	52.12
A6061-13.2%CuO-1%SiO_2_	38.45109	37892	91.21

## Data Availability

Not applicable.

## References

[1] Ahmad A., Lajis M.A., Yusuf N.K., Wagiman A. (2016). Hot Press Forging as The Direct Recycling Technique of Aluminium—A Review. ARPN J. Eng. Appl. Sci..

[2] Birbilis N., Hinton B. (2011). Fundamentals of Aluminium Metallurgy.

[3] Rady M., Mustapa M., Harimon M., Ibrahim M., Shamsudin S., Lajis M., Wagiman A., Msebawi M., Yusof F. (2019). Effect of hot extrusion parameters on microhardness and microstructure in direct recycling of aluminium chips. Mater. Werkst..

[4] Lee C.J., Murakami R.-I., Suh C.M. (2010). Fatigue properties of aluminum alloy (a6061-t6) with ultrasonic nano-crystal surface modification. Int. J. Mod. Phys. B.

[5] Hamzah M.Q., Mezan S.O., Tuama A.N., Jabbar A.H., Agam M.A. (2018). Study and Characterisation of Polystyrene/Titanium Dioxide Nanocomposites (PS/TiO_2_ NCs) for Photocatalytic Degradation Application: A Review. Int. J. Eng. Technol..

[6] Güley V., Güzel A., Jäger A., Ben Khalifa N., Tekkaya A., Misiolek W. (2013). Effect of die design on the welding quality during solid state recycling of AA6060 chips by hot extrusion. Mater. Sci. Eng. A.

[7] Mahdi A.S., Mustapa M.S., Latif N.A., Ab Kadir M.I., Samsi M.A. (2016). Heat treatment for an recycling aluminum aa6061 using milling process at various holding aging time. Int. J. Eng. Technol..

[8] Hussein Rady M., Mustapa M.S., Wagiman A., Al-Alimi S., Shamsudin S., Lajis M.A., Mansor M.N., Harimon M.A. (2020). Effect of the heat treatment on mechanical and physical properties of direct recycled aluminium alloy (AA6061). Int. J. Integr. Eng..

[9] Shamsudin S., Lajis M., Zhong Z. (2016). Solid-state recycling of light metals: A review. Adv. Mech. Eng..

[10] Safiuddin M., Jumaat M.Z., Salam M.A., Islam M.S., Hashim R. (2010). Utilisation of solid wastes in construction materials. Int. J. Phys. Sci..

[11] Allaker R.P. (2012). Nanoparticles and the Control of Oral Biofilms.

[12] Zhu H., Dong K., Huang J., Li J., Wang G., Xie Z. (2014). Reaction mechanism and mechanical properties of an aluminum-based composite fabricated in-situ from Al–SiO_2_ system. Mater. Chem. Phys..

[13] Huo H., Woo K.D. (2006). In situ synthesis of Al_2_O_3_ particulate-reinforced Al matrix composite by low temperature sintering. J. Mater. Sci..

[14] Issa H.K., Taherizadeh A., Maleki A., Ghaei A. (2017). Development of an aluminum/amorphous nano-SiO_2_ composite using powder metallurgy and hot extrusion processes. Ceram. Int..

[15] Rady M.H., Mahdi A.S., Mustapa M.S., Shamsudin S., Lajis M.A., Msebawi M.S., Siswanto W.A., Al Alimi S. (2019). Effect of Heat Treatment on Tensile Strength of Direct Recycled Aluminium Alloy (AA6061). Mater. Sci. Forum.

[16] Sabbar H.M., Shamsudin S., Abbas M.A., Msebawi M.S., Mustapa M.S., Lajis M.A., Rady M.H., Al Alim S. (2020). Study on the Wear Influence for Recycled AA6061 Aluminum/Al_2_O_3_ Utilizing the Face Central-Full Factorial Technique (FCFFT). Advances in Material Sciences and Engineering.

[17] Cai Z., Zhang C., Wang R., Peng C., Wu X. (2016). Effect of copper content on microstructure and mechanical properties of Al/Sip composites consolidated by liquid phase hot pressing. Mater. Des..

[18] Raju P.V.K., Rajesh S., Rao J.B., Bhargava N. (2018). Tribological behavior of Al-Cu alloys and innovative Al-Cu metal matrix composite fabricated using stir-casting technique. Mater. Today Proc..

[19] Ferguson J., Aguirre I., Lopez H., Schultz B.F., Cho K., Rohatgi P.K. (2014). Tensile properties of reactive stir-mixed and squeeze cast Al/CuOnp-based metal matrix nanocomposites. Mater. Sci. Eng. A.

[20] Hassanzadeh-Aghdam M., Mahmoodi M., Ansari R. (2018). A comprehensive predicting model for thermomechanical properties of particulate metal matrix nanocomposites. J. Alloy. Compd..

[21] Hitziger M., Ließ M. (2014). Comparison of Three Supervised Learning Methods for Digital Soil Mapping: Application to a Complex Terrain in the Ecuadorian Andes. Appl. Environ. Soil Sci..

[22] Jamson A.H., Merat N., Carsten O.M., Lai F.C. (2013). Behavioural changes in drivers experiencing highly-automated vehicle control in varying traffic conditions. Transp. Res. Part C Emerg. Technol..

[23] Suzuki Y., Hino H., Hawai T., Saito K., Kotsugi M., Ono K. (2020). Symmetry prediction and knowledge discovery from X-ray diffraction patterns using an interpretable machine learning approach. Sci. Rep..

[24] Papadopoulos S., Azar E., Woon W.L., Kontokosta C. (2017). Evaluation of tree-based ensemble learning algorithms for building energy performance estimation. J. Build. Perform. Simul..

[25] McWilliams C.J., Lawson D.J., Santos-Rodriguez R., Gilchrist I.D., Champneys A., Gould T.H., Thomas M.J., Bourdeaux C.P. (2019). Towards a decision support tool for intensive care discharge: Machine learning algorithm development using electronic healthcare data from MIMIC-III and Bristol, UK. BMJ Open.

[26] Hajjem A., Bellavance F., Larocque D. (2014). Mixed-effects random forest for clustered data. J. Stat. Comput. Simul..

[27] Chiba R., Yoshimura M. (2015). Solid-state recycling of aluminium alloy swarf into c-channel by hot extrusion. J. Manuf. Process..

[28] Kondoh K., Luangvaranunt T., Aizawa T. (2002). Solid-State Recycle Processing for Magnesium Alloy Waste via Direct Hot Forging. Mater. Trans..

[29] Fann K.-J., Chen C.-C. (2017). Grain Size in Aluminum Alloy 6061 under Hot Ring Compression Test and after T6 Temper. Appl. Sci..

[30] Ab Rahim S., Lajis M., Ariffin S. (2015). A Review on Recycling Aluminum Chips by Hot Extrusion Process. Procedia CIRP.

[31] Majdi H., Razaghian A., Emamy M., Motallebi N. (2017). The effects of Cu addition and solutionising heat treatment on the microstructure and wear properties of hot-extruded Al–Mg_2_Si eutectic alloy. Adv. Mater. Process. Technol..

[32] Rachman A., Ratnayake R.C. (2019). Machine learning approach for risk-based inspection screening assessment. Reliab. Eng. Syst. Saf..

[33] Li S., Zhang X. (2020). Research on orthopedic auxiliary classification and prediction model based on XGBoost algorithm. Neural Comput. Appl..

[34] Injadat M., Moubayed A., Nassif A.B., Shami A. (2020). Systematic ensemble model selection approach for educational data mining. Knowl. -Based Syst..

[35] Eberhart R.C., Shi Y. (1998). Comparison between genetic algorithms and particle swarm optimization. International Conference on Evolutionary Programming.

[36] Sun J., Yang Y., Wang Y., Wang L., Song X., Zhao X. (2020). Survival Risk Prediction of Esophageal Cancer Based on Self-Organizing Maps Clustering and Support Vector Machine Ensembles. IEEE Access.

[37] Das S., Chandrasekaran M., Samanta S. (2018). Comparison of Mechanical properties of AA6061 reinforced with (SiC/B_4_C) micro/nano ceramic particle reinforcements. Mater. Today Proc..

[38] Elanchezhian C., Ramanth B., Bhaskar G.B., Vivekanandhan M. (2019). An Investigation of the Mechanical Properties of HybridComposites in Applications of Automotive Industry. Mater. Today Proc..

[39] Gayathri J., Elansezhian R. (2020). Influence of dual reinforcement (nano CuO + reused spent alumina catalyst) on microstructure and mechanical properties of aluminium metal matrix composite. J. Alloy. Compd..

[40] Pandiyan A., Kumar G.A., Ranganthan S., Madhu S. (2019). Optimization of wear performance on aluminium die cast A360-M1 master alloy using response surface method. Mater. Today Proc..

[41] Singh B., Kumar J., Kumar S. (2014). Experimental Investigation on Surface Characteristics in Powder-Mixed Electrodischarge Machining of AA6061/10%SiC Composite. Mater. Manuf. Process..

[42] Shamsudin S., Zhong Z.W., Ab Rahim S.N., Lajis M.A. (2017). The influence of temperature and preheating time in extrudate quality of solid-state recycled aluminum. Int. J. Adv. Manuf. Technol..

[43] Sahoo B., Narsimhachary D., Paul J. (2019). Tribological characteristics of aluminium-CNT/graphene/graphite surface nanocomposites: A comparative study. Surf. Topogr. Metrol. Prop..

[44] Fogagnolo J.B., Ruiz-Navas E.M., Simón M.A., Martinez M.A. (2003). Recycling of aluminium alloy and aluminium matrix composite chips by pressing and hot extrusion. J. Mater. Process. Technol..

[45] Paulraj G.M., Parth S., Debraj B. (2018). Hydrothermal Pretreatment of Tender Coconut Coir and Optimization of Process Parameters Using Response Surface Methodology. Urbanization Challenges in Emerging Economies: Energy and Water Infrastructure.

[46] Ravikumar M., Reddappa H., Suresh R. (2018). Aluminium Composites Fabrication Technique and Effect of Improvement in Their Mechanical Properties—A Review. Mater. Today Proc..

[47] Jin L., Xue J., Zhang Z., Hu Y. (2018). Effects of Thermal Frequency on Microstructures, Mechanical and Corrosion Properties of AA6061 Joints. Appl. Sci..

[48] Sharma N., Khanna R., Singh G., Kumar V. (2017). Fabrication of 6061 aluminum alloy reinforced with Si_3_N_4_/n-Gr and its wear performance optimization using integrated RSM-GA. Part. Sci. Technol..

[49] Reddy P.V., Prasad P.R., Krishnudu D.M., Goud E.V. (2019). An Investigation on Mechanical and Wear Characteristics of Al 6063/TiC Metal Matrix Composites Using RSM. J. Bio- Tribo-Corros..

[50] Khamis S., Lajis M., Albert R. (2015). A Sustainable Direct Recycling of Aluminum Chip (AA6061) in Hot Press Forging Employing Response Surface Methodology. Procedia CIRP.

[51] Rajakumar S., Muralidharan C., Balasubramanian V. (2011). Predicting tensile strength, hardness and corrosion rate of friction stir welded AA6061-T6 aluminium alloy joints. Mater. Des..

[52] Camposeco-Negrete C. (2015). Optimization of cutting parameters using Response Surface Method for minimizing energy consumption and maximizing cutting quality in turning of AISI 6061 T6 aluminum. J. Clean. Prod..

[53] Adam S.P., Alexandropoulos S.-A.N., Pardalos P.M., Vrahatis M.N. (2019). No free lunch theorem: A review. Approx. Optim..

[54] Sathyajith S., Kalainathan S. (2012). Effect of laser shot peening on precipitation hardened aluminum alloy 6061-T6 using low energy laser. Opt. Lasers Eng..

